# Morphological and Developmental Features of Stone Cells in *Eriobotrya* Fruits

**DOI:** 10.3389/fpls.2022.823993

**Published:** 2022-01-27

**Authors:** Shoukai Lin, Dahe Lin, Bisha Wu, Shiwei Ma, Shengfeng Sun, Ting Zhang, Wenting Zhang, Yunlu Bai, Qiong Wang, Jincheng Wu

**Affiliations:** ^1^College of Environmental and Biological Engineering, Putian University, Putian, China; ^2^Fujian Provincial Key Laboratory of Ecology-Toxicological Effects & Control for Emerging Contaminants, Putian University, Putian, China; ^3^College of Horticulture, South China Agricultural University, Guangzhou, China

**Keywords:** *Eriobotrya* plants, stone cells, fruits, morphological and developmental features, trait transmission

## Abstract

Some members of the *Rosaceae* family, particularly pear, contain stone cells in their fruits. Although stone cells in pear fruits are well studied, relatively little attention has been given to loquat stone cells. Only a few reports have suggested a relationship between stone cell traits and storage and transport tolerance of loquat fruits. Previously, we generated the variety JT8 from the interspecific hybrid of the loquat cultivar Jiefangzhong (JFZ; *Eriobotrya japonica* Lindl. cv. Jiefangzhong, female parent) and wild Taiwanese loquat (TL; *E. deflexa* Nakai, male parent). The JT8 fruits had a granular feel, similar to that of pear fruits, due to the presence of stone cells. In this study, the shape, size, development, and distribution dynamics of stone cells of *Eriobotrya* plants were thoroughly investigated. The results showed that loquat stone cells are brachysclereids and often contain typical branching pits. Loquat stone cells were distributed as both single stone cells and in stone cell clusters (SCCs), and the density of the stone cells near the core was higher than that near the peel. Stone cell density first increased and then decreased during fruit development. These traits noted in *Eriobotrya* were very similar to those observed in pear, indicating a close relationship between loquat and pear. Moreover, the contents, density dynamics, and aggregation traits of stone cells of the interspecific hybrid JT8 were derived from the male parent (TL). Transgressive segregation was likely exhibited in the content of stone cells and the size of the SCCs. More specifically, the content of stone cells reached 1.61% (w/w). In extreme cases, SCCs of JT8 exceeded 1,000 μm in length and 500 μm in width. This demonstrated that stone cell traits could be transmitted from parent to progeny through interspecific hybridization. The density dynamics of stone cells in two loquat cultivars with different storage and transport tolerances were also investigated, which indicated that the cultivar with more stone cells was more tolerant to storage and transport. We suggest that wild loquat genetic resources containing stone cells in *Eriobotrya* plants can be used to gradually improve the storage and transport tolerance of loquat fruits.

## Introduction

Loquat (*Eriobotrya japonica* Lindl.), a characteristic evergreen fruit tree species originating from China, is now distributed in more than 30 countries, mainly in East Asian, South Asian, and coastal Mediterranean countries. Loquat fruits are not easy to store and transport, and the period during which fresh fruits are available is short, which restricts the improvement of the planting efficiency of loquat (Lin et al., 2018). Some kinds of *Rosaceae* fruits contain numerous stone cells, such as pear and haw fruits, which make a distinct impression to many people. Although careful researchers have noted that some loquat cultivars also contain stone cells distributed within their fruits, little attention has been given to stone cells in loquat, which also belongs to *Rosaceae*. Stone cells were larger and more abundant in the storage- and transport-tolerant cultivars Jiefangzhong, Wugongbai, and Ruoyangqing and smaller and rarer in the storage- and transport-susceptible cultivars Changhong No. 3, Baili, and Baisha, suggesting that stone cells exist in ripe fruits of some loquat varieties and are related to the storage and transport tolerance of loquat fruits (Lin et al., 2009; Zhu et al., 2018).

Stone cells (or sclereids) are sclerenchyma cells formed by the secondary deposition of lignin on the primary cell wall of parenchyma cells. Their development is closely related to the synthesis, transfer, and deposition of lignin. Stone cells can be distributed as single cells or as aggregates known as stone cell clusters (SCCs; Nii et al., 2008). To the best of our knowledge, many *Rosaceae* fruits contain stone cells, including those of *Pyrus* spp., *Eriobotrya japonica*, *Chaenomeles sinensis*, *Crataegus pinnatifida*, *Prunus mume*, *Prunus armeniaca*, *Prunus salicina*, and so forth (Huang et al., 2005; Sun and Li, 2006; Lin et al., 2009; Pan, 2011; Wu et al., 2013). In particular, the fruits of pear cultivars contain a large number of stone cells that significantly affect the quality of their fruits, thereby attracting much attention and making these cultivars model plants for the study of fruit stone cells (Wu et al., 2013). The literature thus far has shown that most of the studies on fruits containing stone cells are few and unthorough, except those pertaining to pear fruits. For instance, when describing the overall quality of hawthorn fruits, it was only mentioned that there were few stone cells (Pan, 2011).

In pear, secondary cell wall thickening and lignin deposition are key steps in the developmental process from parenchyma cells to stone cells. First, lignin is deposited along the apical angles of the primary cell wall and then gradually diffuses, which leads to uneven thickening of the cell walls. With the thickening of the secondary cell wall, the cell contents gradually shrink to the cell center. Eventually, the cell contents disappear, and the entire cell is completely composed of secondary cell wall structures (Choi and Lee, 2013; Zhao et al., 2013). Pear stone cells are brachysclereids that are characterized by thickened secondary cell walls with severe lignification, often with branching pits (Jin et al., 2013). The cell wall components of pear stone cells contain large amounts of lignin, cellulose, and hemicellulose (xylans), while the parenchyma cells of pear fruits contain abundant pectin (Brahem et al., 2017). The distribution of stone cells in pear flesh is not uniform and changes significantly in different growth stages (Choi and Lee, 2013). During fruit development, the density of stone cells first increased and then decreased, and the density of stone cells in different pear varieties reached its peak at different times (Zhang et al., 2017). Nii et al. (2008), Choi and Lee (2013), and Zhang et al. (2017) reported dynamic changes in stone cell distribution during the developmental process of pear fruits. The density of stone cells is higher near the core than near the peel. In addition, the number and size of stone cell clusters near the core are often higher than those near the peel (Tao et al., 2009). Different species and cultivars have diverse stone cell traits, such as content, shape, and size (Tao et al., 2009). When fruits can be used for traditional Chinese medicine, stone cell morphology is often used as one of the bases for the identification of medicinal varieties, such as *Crataegus pinnatifida* and *Prunus mume* (Huang et al., 2005; Sun and Li, 2006). Cao et al. (2010) found that the content of stone cells in pear pulp differed among 304 cultivars and interspecific hybrids. Tian et al. (2011) found that the size of SCCs in pear pulp differed among 319 cultivars and interspecific hybrids. Different stone cell traits are related to the texture and quality of pear fruits (Cao et al., 2010; Tian et al., 2011). There is a positive correlation between the content of pear stone cells and the firmness, adhesiveness, and chewiness of pear pulps (Choi et al., 2007; Liu et al., 2020).

In contrast, the fruits of loquat cultivars were similar to pear cultivars in the shape of stone cells, which belong to brachysclereids (Lin et al., 2009; Zhu et al., 2018). A series of recent studies reported the composition and development of loquat stone cells. It was found that loquat stone cells contained lignin, cellulose, and hemicellulose, the cell corner (CC) and middle lamella (ML) deposited only lignin and pectin, and parenchyma cells contained almost no lignin (Zhu et al., 2018, 2019). During the development of loquat stone cells, lignin and cellulose gradually filled stone cells, while pectin mainly filled the CC and ML. Loquat stone cells contained abundant lignin functional groups of coniferaldehyde and coniferyl alcohol (Huang et al., 2019). Stone cells and vascular bundles were the active areas of lignin deposition. Cyclic stone cells deposited lignin in both the inner and outer layers. When stone cells are fully filled with lignin, the outer layer of stone cells can still continue to deposit lignin so that adjacent parenchyma cells can also start to deposit lignin (Zhu et al., 2021). Therefore, some stone cells can be observed alone and surrounded by parenchyma cells. On the other hand, some parenchyma cells around stone cells also accumulate lignin, which later becomes stone cells and eventually forms stone cell clusters (Huang et al., 2019; Zhu et al., 2021). Interestingly, the newly synthesized lignin in loquat pulp was specifically deposited in the CC and ML of parenchyma cells around vascular bundles during storage, thus forming a network structure (Zhu et al., 2021).

The above studies indicated that pear stone cell traits were closely related to the texture and quality of pear fruits, which has become an important index in pear breeding. In contrast, stone cell traits of loquat fruits are not well known, and some reports involving loquat stone cells suggested the existence of a certain relationship between stone cell traits and storage and transport tolerance in loquat fruits. Our former research work produced the hybrid variety JT8 from the interspecific hybrid of the common yellow-fleshed loquat cultivar Jiefangzhong (JFZ; *Eriobotrya japonica* Lindl. cv. Jiefangzhong, female parent) and wild Taiwanese loquat (TL; *E. deflexa* Nakai, male parent). It was found that the fruits of JT8 obviously had a stone cell taste similar to that of pear fruits. Although stone cell traits have potential application value to improve the storage and transport tolerance of loquat cultivars, the key problem of stone cell trait transmission from parents to progenies in interspecific crosses has not been solved in loquat. Therefore, in this study, the shape, size, development, and distribution dynamics of stone cells of *Eriobotrya* plants were thoroughly studied, and the potential breeding value of stone cell traits in improving the storage and transport tolerance of loquat fruits and the transmission of stone cell traits from parents to progeny in interspecific crosses were discussed.

## Materials and Methods

### Plant Materials and Treatments

The fruits of *Eriobotrya* plants were used as plant materials, including the common loquat cultivars Jiefangzhong (JFZ; *Eriobotrya japonica* cv. Jiefangzhong) and Baili (BL; *E. japonica* cv. Baili), the wild Taiwanese loquat (TL; *E. deflexa* Nakai) and another form of Taiwanese loquat (TLk; *E. deflexa* f. *koshunensis* Nakai), and the following interspecific hybrids: JFZ (♀) × TL (♂) No. 8 (JT8) and JFZ (♀) × TLk (♂) No. 2 (JTk2) and No. 3 (JTk3). The fruits of cultivars JFZ and BL were derived from mature trees located in an outdoor orchard at 25°45' N and 118°55' E (Changtai town, Putian city, Fujian province, China). The fruits of other loquat plants were sampled from mature trees located in Loquat Plants Resource Nursery at 23°16' N and 113°37' E (South China Agricultural University, Guangzhou city, Guangdong province, China). The cultivars JFZ and BL underwent flower thinning at full bloom. Each inflorescence contained 10 ~ 15 fertilized ovaries with no petals and withered flower cores. Wild loquats and interspecific hybrids had few flower inflorescences; therefore, flower thinning was not carried out for these plants. The start of full bloom was defined as 0 DAF (days after full bloom). Three groups of samples were collected separately: the first group was used to determine the stone cell content and consisted of the mature fruits of JFZ, TL, TLk, JT8, JTk2, and JTk3; the second group was used to thoroughly investigate the shape, size, development, and distribution dynamics of stone cells and included the fruits of JFZ and JT8, which were sampled at 14, 28, 42, 56, 70, 84, 98, 112, and 126 DAF, and the fruit of TL, which was sampled on only four occasions at 14, 98, 112, and 126 DAF due to rare occurrence (in some years, no fruit was produced); and the third group was used to study the relationship between storability, fruit hardness, and stone cell development and included fruits of the storage- and transport-tolerant cultivar JFZ and susceptible cultivar BL (Lin et al., 2008), which were sampled at 14, 28, 63, 98, and 126 DAF.

### Determination of the Stone Cell Content

The stone cell content of mature fruits was determined according to Nie’s method with some modifications (Nie et al., 2006). The ripe loquat pulp was cut up and frozen in a freezer (−20°C) for 24 h. After thawing, the pulps were homogenized at 22,000 rpm for 3 min. The homogenized pulps were washed and incubated with 0.8 L distilled water for 3 min, and then, the supernatants were collected. The procedure was repeated several times until there was no sediment. The collected supernatants were passed through filter paper, and the stone cells remained on the filter paper. The collected stone cells were dried and weighed, and the content was calculated as follows: stone cell content (%) = weight of collected stone cells (g DW)/weight of fresh pulp (g FW) × 100.

### Observation of Fruit Cross-Sections With Phloroglucinol-HCl Staining

Fresh loquat fruit was cut along the equatorial plane, and the cross-section was covered with 1 M HCl and incubated for 10 min. After slight drying with absorbent paper, the cross-section was covered with 1% phloroglucinol. After 10 min, the cross-section was observed by a SX-3 stereoscopic microscope (Shanghai Optical Instrument Factory, China) with a 20× microscopic field.

### Observation of Frozen Sections With Phloroglucinol-HCl Staining

The fruit pulp was divided into small pieces of 0.5 mm × 0.5 mm × 0.5 mm and fixed with 4% paraformaldehyde (PFA) overnight. After that, the small pieces were embedded by using O.C.T. compound (SAKURA, Japan). Frozen tissue sections with a thickness of 30 μm were cut by a CM1850 freezing microtome (Leica, Germany) and placed on Superfrost Plus microscope slides (Thermo, United States). The sections were covered with 1 M HCl and incubated for 1 min, and then, an equal volume of 1% phloroglucinol was added. After 10 min, the frozen sections were observed by a DMi8 inverted fluorescence microscope (Leica, Germany) with a 400× microscopic field.

### Data Statistics

The stone cell density of the fruits was calculated as the proportion of the phloroglucinol-HCl stained area to the total area in the equatorial cross-section of the fruit pulp. Statistical analysis was undertaken using ImageJ software. The length and width of stone cells in the microscopic field of the frozen sections were measured. GraphPad Prism 8 (GraphPad Software, United States) was used for data statistics and plotting. One-way ANOVA was used to test significant differences among three columns, while Student’s *t*-test was used for two columns.

## Results

### Determination of Stone Cell Content in the Common and Wild Loquat Parents and Interspecific Hybrids

**Figure 1:**
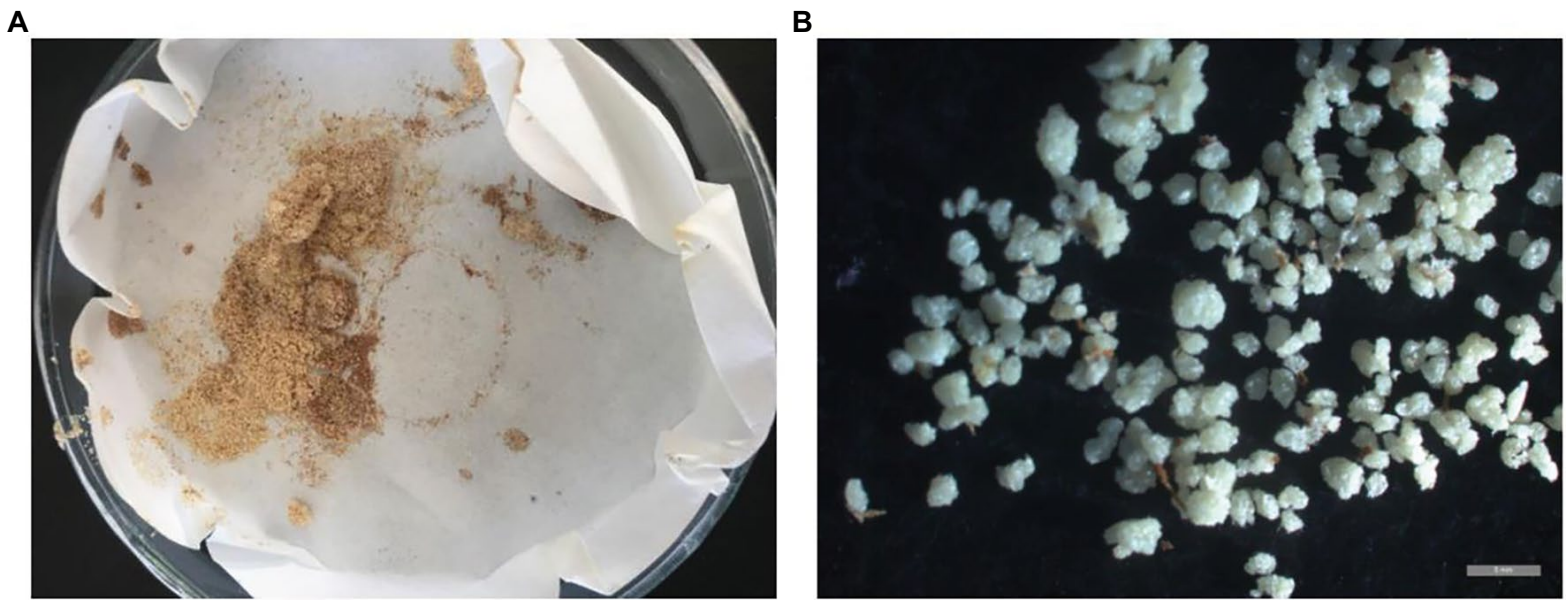
The stone cells extracted from JT8. **(A)** The appearance of stone cells. **(B)** The microscopic morphology of stone cells. The gray bar represents 1 mm.

As shown in Table 1, the stone cell content of the common loquat JFZ was very low (0.03%), while the contents of the wild loquat TL (1.59%) and TLk (0.95%) were at least one order higher than that of JFZ. The interspecific hybrid JT8 (from JFZ × TL) had a high stone cell content, of which the mass ratio was up to 1.61%, higher than that of the male parent TL. In addition, high stone cell contents were also observed in the interspecific hybrids JTk2 and JTk3 (from JFZ × TLk), with mass ratios of 0.79 and 0.96%, respectively. The results showed that the high stone cell contents of the interspecific hybrids probably came from the male parent wild loquat, which could exhibit transgressive inheritance. Microscopic observation of stone cells extracted from the fruits of the interspecific hybrid JT8 revealed many large stone cells or stone cell clusters (Figure 1). This may be the main reason for the granular taste of JT8 fruits.

**Table 1:** Stone cell contents in different loquat plants.

Loquat genotypes	Pulp weight (g)^*^	Stone cell weight (g)	Proportion (w/w)
JFZ	30.83	0.01	0.03%
TL	32.12	0.51	1.59%
TLk	36.58	0.34	0.95%
JT8	34.79	0.56	1.61%
JTk2	35.25	0.28	0.79%
JTk3	30.11	0.29	0.96%

* The total weight of pulps used to determine stone cell content.

### Stone Cell Distribution Traits in the Common and Wild Loquat Parents and Interspecific Hybrids During Fruit Development

**Figure 2:**
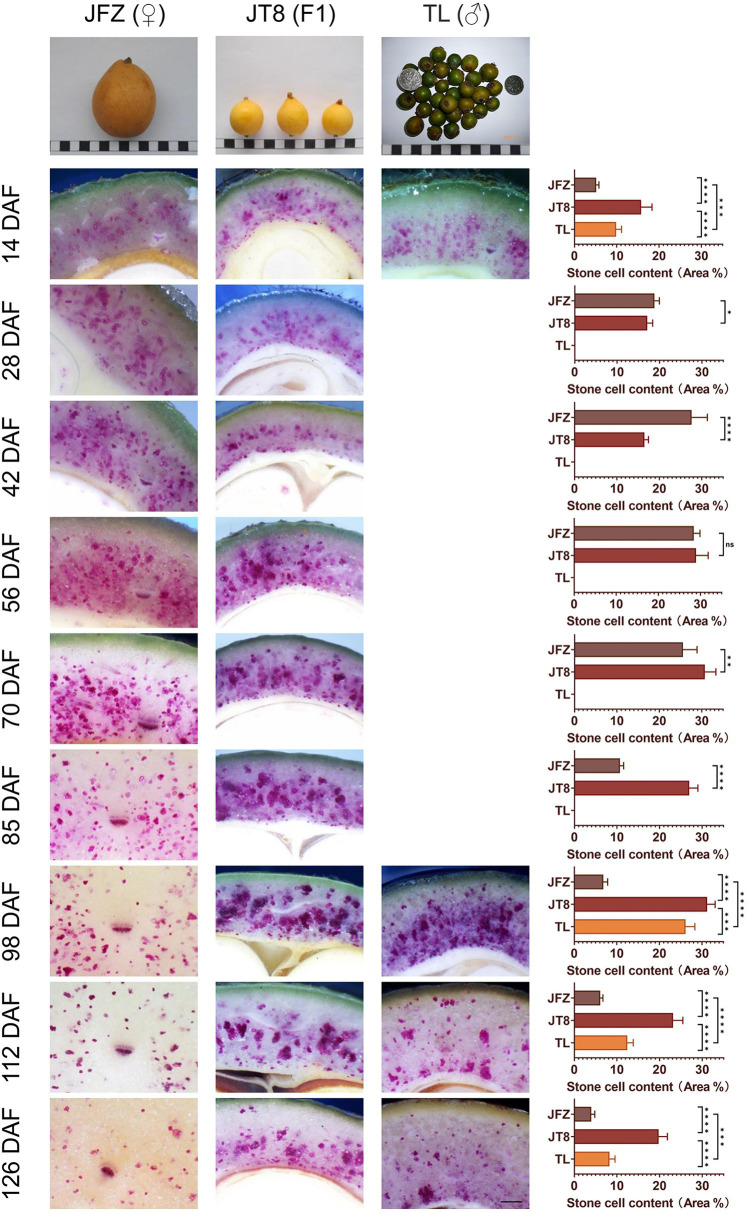
Phloroglucinol-HCl staining and microscopic observation of transverse sections of fruit pulp from three loquat plants. Due to the few samples in wild loquat TL, only four developmental stages were examined, including 14, 98, 112, and 126 DAF. Three fruit images at the top of the figure show the mature fruit size, shape, and color of three loquat plants; in particular, the color of mature TL fruits was brown green. The width of each black or white box represents 1 cm; the black bar at lower right represents 500 μm; bar charts show the stone cell density; and “ns” represents that there is no significant difference, “*” represents value of *p* < 0.05, “**” represents value of *p* < 0.01, “***” represents value of *p* < 0.001, and “****” represents value of *p* < 0.0001.

Cross-sections of JFZ, TL, and JT8 fruits with phloroglucinol-HCl staining were observed and are displayed in Figure 2. The observation of TL was only performed at four time points at 14, 98, 112, and 126 DAF because of its few fruits. The fruit size of JFZ was significantly larger than those of TL and JT8, and the fruit size of JT8 was between those of the two parents and closer to the male parent TL. At 14 DAF, the outlines of numerous cells were light purple in the fruits of three *Eriobotrya* plants, indicating that lignin deposition and secondary cell wall thickening had occurred and that the formation of stone cells had begun before 14 DAF. After that, the stained cell outline became increasingly thick, the color increasingly deepened, and an increasing number of entire cells became dark purple. The results showed that with the development of fruits, lignin continued to fill in the sclerenchyma cells until the protoplasts disappeared and the stone cells were completely developed. The stone cell density of the JFZ rapidly increased from 5.16% at 14 DAF to 27.58% at 42 DAF, reached a maximum value of 28.40% at 56 DAF, and then slightly decreased to 25.24% at 70 DAF. It then rapidly decreased to 10.68% at 85 DAF, after which it gradually declined to 3.98% until the mature stage at 126 DAF. The stone cells tended to aggregate into small SCCs in the JFZ. The stone cell density of the wild loquat TL was significantly higher than that of JFZ and lower than that of JT8 at the four sampled time points. The stone cells increased from 9.85% at 14 DAF to 26.12% at 98 DAF with a dense and wide distribution and then decreased to 8.25% at 126 DAF, at which time many large stone cell clusters were still apparent. In the interspecific hybrid JT8, the stone cell density also presented a trend of early increase and later decrease. The stone cell density increased from 15.77% at 14 DAF to a maximum value of 31.19% at 98 DAF, followed by a decrease to 19.77% at 126 DAF. The time of peak stone cell density in JT8 may be the same as that of the male parent TL and later than that of the female parent JFZ. The dense distribution of stone cells and many large SCCs also appeared in JT8, similar to the male parent TL. The density and aggregation traits of stone cells of JT8 were similar to those of the male parent TL, suggesting that these stone cell traits were derived from the male parent. Interestingly, the density of stone cells and large SCCs was obviously higher than that of the two parents, indicating that some traits of stone cells may exhibit transgressive inheritance. In fruits at 126 DAF, the density of stone cells was higher near the core than near the peel, and the SCCs near the core were larger than those near the peel, while the distribution of stone cells in TL near the peel was higher than that in JT8 and JFZ, which indicated that some traits of stone cells could be inherited from the female parents. Interestingly, no obvious stone cells could be found in the peel tissues of the three *Eriobotrya* plants. In brief, the key finding was that stone cell traits could be transmitted from parents to progenies in interspecific crosses and that the transgressive inheritance of stone cell traits might occur.

### Morphological and Developmental Traits of Stone Cells in Common and Wild Loquat Parents and Interspecific Hybrids

**Figure 3:**
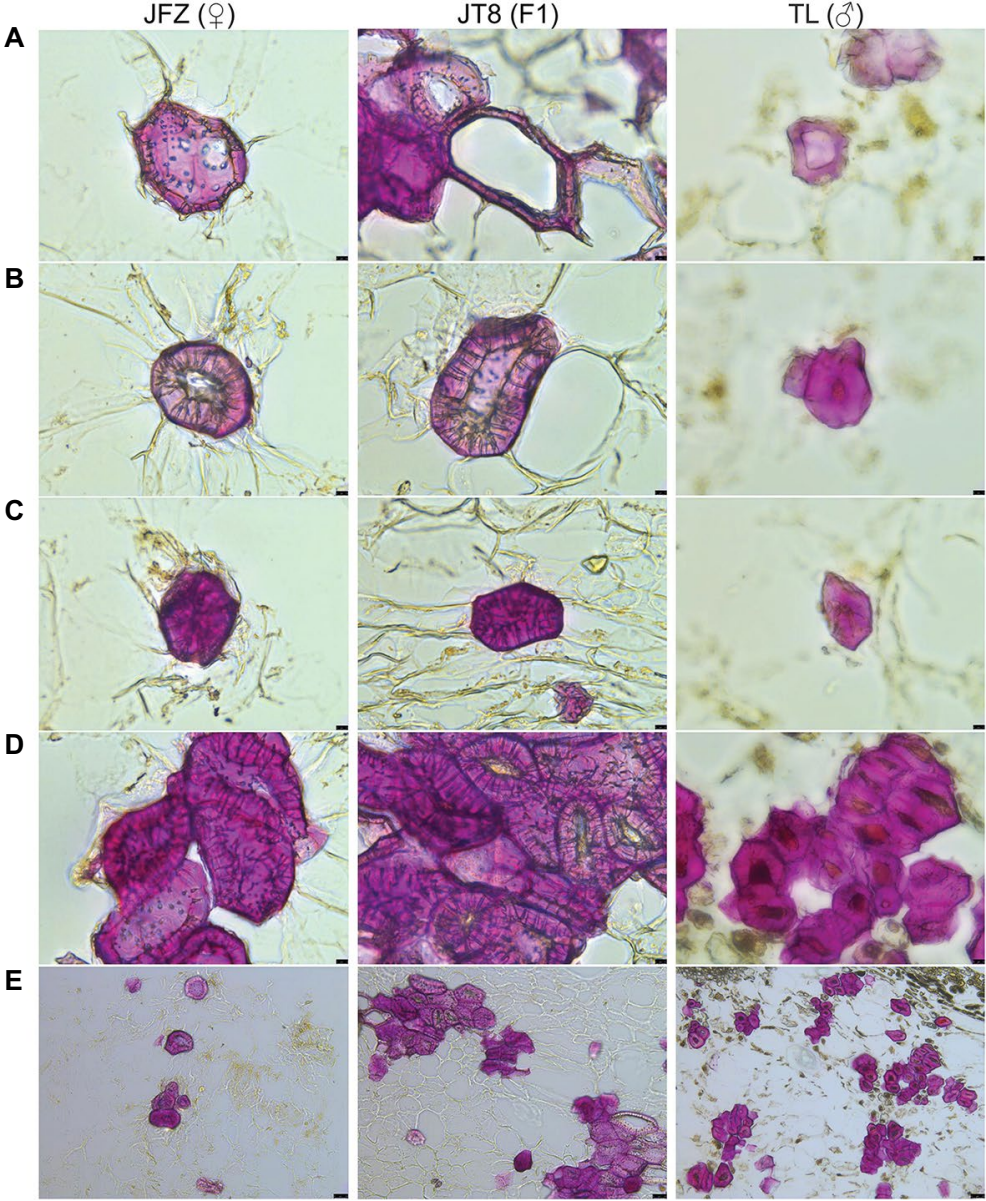
Phloroglucinol-HCl staining and microscopic observation of freezing microtome sections of fruit pulp taken from three loquat plants at 126 DAF. **(A)** The secondary cell wall of sclereid primordium cells starts to thicken (400×). **(B)** The sclereid primordium cell shows a continuously thickened secondary cell wall and shrunken protoplasm (400×). **(C)** The typical stone cell is filled by secondary cell wall structures without protoplasm (400×). **(D)** Stone cell clusters (400×). **(E)** Stone cell clusters (100×). The black bar in the lower right represents 10 μm in **(A,B)** and 50 μm in **(E)**.

**Figure 4:**
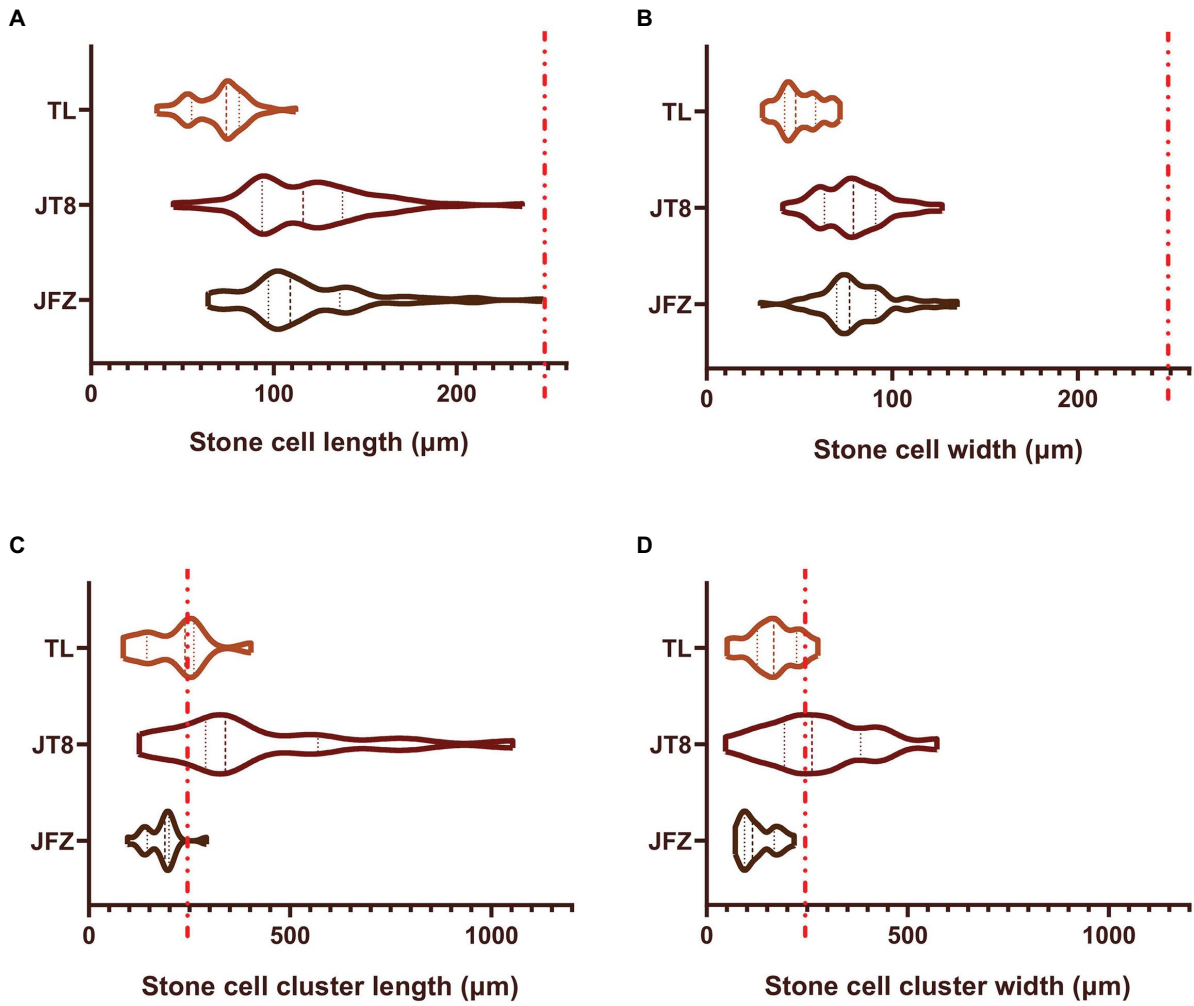
The sizes of stone cells and stone cell clusters in freezing microtome sections of fruit pulp collected at 126 DAF from three loquat plants as determined through phloroglucinol-HCl staining and microscopic observation. Violin plots are used to display the distribution of data sets, and similar violin shapes represent similar data distributions. The thick dashed lines represent the median values, and the two thin dashed lines represent the first and third quartiles. The wider parts of the violin parts correspond to higher probabilities of observed values, while the narrower parts correspond to lower probabilities. Longer and sharper ends of the violin plots correspond with more outliers. The red-dashed line indicates 250 μm. **(A)** Stone cell length; **(B)** Stone cell width; **(C)** Stone cell cluster length; **(D)** Stone cell cluster width.

The stone cells of three *Eriobotrya* plants are brachysclereids, and their developmental processes are almost the same. The development of stone cells was accompanied by the thickening of secondary cell walls and the shrinkage of protoplasts until the formation of typical stone cells filled with secondary cell walls occurred, and the protoplasts often disappeared. The pits of stone cells were thicker and clear with branching in the JFZ but thinner and unclear in the TL. There were more pairs of interlinked pits between adjacent stone cells in the JFZ and fewer in the TW. The shape and size of pits in JT8 were similar to those in the female parent JFZ (Figures 3A–C). The statistical results showed that the length and width of stone cells and SCCs were not evenly distributed and exhibited great variation. The stone cell length and width of JFZ and JT8 varied widely, while those of TL did not. The median and quartile values of the length and width of stone cells between JFZ and JT8 were similar and were significantly greater than those of TL (Figures 4A,B). The results indicated that the traits of shape and size of stone cells tended to be inherited from the female parent JFZ. In addition, the density of stone cells in the JFZ was sparse, there were only a few stone cells aggregated in single SCCs, while the density of stone cells in the TL was higher, and there were many stone cells aggregated in single SCCs (Figures 3D,E). The statistical results showed that the ranges of stone cell length and width of the two parents were small, while those of JT8 were very dispersed. The median and quartile values of the length and width of TL stone cells were greater than those of JFZ stone cells, and both of them were smaller than those of JT8 stone cells. The length of most JT8 SCCs, a small proportion of TL SCCs and some JFZ SCCs, and the width of more than half of JT8 SCCs and a small proportion of TL SCCs were greater than 250 m (Figures 4C,D). The results illustrated that although the size of TL stone cells was small, the stronger aggregation of TL stone cells led to larger-sized SCCs in the TL than in the JFZ. However, the aggregation of stone cells in JT8 tended to be derived from the male parent TL, and the size of stone cells tended to be inherited from the female parent JFZ, which resulted in the size of JT8 SCCs exhibiting transgressive inheritance. It is worth noting that in the fruits at 126 DAF, newborn sclereid primordium cells with thin secondary cell walls could be observed in JFZ and JT8 but were rare in TL. This result suggested that JFZ and JT8 fruits may be sustained to produce new stone cells, while TL may be incapable in this regard after a certain time point. These results also supported our previous statement that stone cell traits could be transmitted from parents to progenies and between different species, and that the transgressive inheritance of stone cell traits might occur.

### The Distribution of Stone Cells in Two Loquat Cultivars With Different Storage and Transport Tolerances During Fruit Development

**Figure 5:**
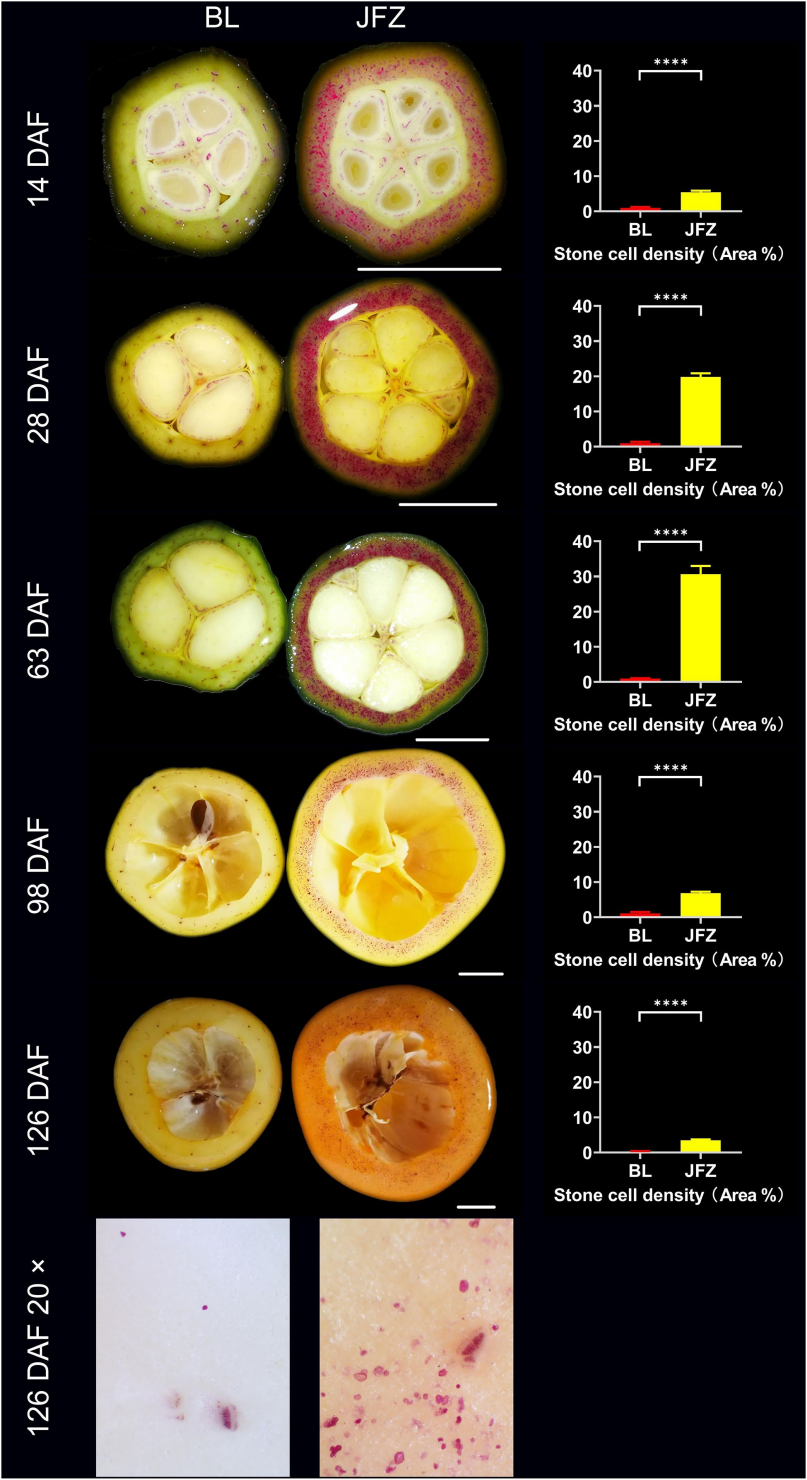
Phloroglucinol-HCl staining and microscopic observation of transverse sections of fruit pulp from white and yellow loquat fruits. DAF: Days after full bloom; BL: White-fleshed loquat fruit (*Eriobotrya japonica* Lindl. cv. Baili); JFZ: Yellow-fleshed loquat fruit (*Eriobotrya japonica* Lindl. cv. Jiefangzhong); and 20×: 20× stereoscopic microscopy observations. Bar charts show the stone cell density; “****” represents value of *p* < 0.0001.

BL was susceptible to storage and transport, its pulp texture was very fine, while JFZ was tolerant to storage and transport, and its pulp texture was coarse (Lin et al., 2008). As shown in Figure 5, the stone cell density in BL fruit remained at approximately 1.00% from 14 DAF to 98 DAF and then decreased to 0.32% at 126 DAF. In contrast, the density of stone cells in the JFZ first increased from 5.39% at 14 DAF to 30.67% at 63 DAF and then decreased to 3.51% at 126 DAF. Undoubtedly, the number of stone cells in the JFZ was much higher than that in the BL. The results showed that stone cell traits were correlated with the storage and transport tolerance and pulp texture of loquat fruits and that higher stone cell content and density might be beneficial to enhance the storage and transport tolerance while worsening the taste of loquat fruits. A 20× stereoscopic microscope revealed that many stone cells were scattered in 126 DAF JFZ fruits, and some of them were aggregated into small SCCs, while only a few single stone cells were found in 126 DAF BL fruits. This may be the reason why JFZ has a less desirable taste than BL.

## Discussion

Stone cells (or sclereids) are sclerenchyma cells characterized by thickening and lignified cell walls (Nii et al., 2008; Zhang et al., 2017). Stone cells can be classified into five types according to their morphology: brachysclereids, macrosclereids, osteosclereids, astrosclereids, and trichosclereids (Zhao and Zhu, 2014). Stone cells are generally believed to have greater hardness than parenchyma cells and thus could serve a supporting function (Brahem et al., 2017). Moreover, stone cells have also been found to act as a physical defense against white pine weevils in Sitka spruce (Whitehill et al., 2016). At present, most studies on stone cells have been carried out in pear fruits, while only a few have mentioned loquat (Lin et al., 2009; Zhu et al., 2018). In this study, the shape, size, development, and distribution dynamics of fruit stone cells of *Eriobotrya* plants were thoroughly studied. The stone cell traits of *Eriobotrya* plants were highly similar to those of pear, especially the following traits. Pear stone cells are brachysclereids and often include branching pits (Choi and Lee, 2013). Pear stone cells can be found as single cells or as SCCs, and they are usually distributed as SCCs (Wu et al., 2013; Brahem et al., 2017). Some pairs of interlinked pits could often occur between adjacent stone cells. The secondary cell walls of some pear parenchyma cells are thickened first to form sclereid primordium cells, and then the secondary cell walls are continuously thickened until the protoplasm disappears and the cell fills with secondary cell wall structures. The stone cell development is then complete (Jin et al., 2013; Zhao et al., 2013). With the development of pear fruits, the rate of stone cell production was higher than that of fruit expansion, and therefore, the density of stone cells increased until the peak. Subsequently, the rapid expansion of parenchyma cells resulted in a fruit expansion rate greater than the stone cell production rate, resulting in a continuous decrease in stone cell density. The time for stone cells to reach the peak distribution density varied among different pear varieties. In addition, the distribution of stone cells in the pear pulp was not uniform. The density of stone cells was higher and the size of the SCCs larger near the core, but the opposite was found near the peel (Nii et al., 2008; Choi and Lee, 2013; Zhang et al., 2017). These similar traits between loquat and pear stone cells indicated the existence of a relationship between loquat and pear, which may provide new ideas for the study of the relationship and evolution among the fruit trees of *Rosaceae*. Moreover, due to the highly similar features of stone cell traits between loquat and pear fruits, the relevant research results of pear stone cells can be used as an important reference for the study of loquat stone cells.

The pear-like granular taste is hardly found in the fruit of loquat cultivars, so there is not much consumer concern about whether loquat fruits contain stone cells. Prof. Lin Shunquan, one of the authors of this paper, collected 26 species of *Eriobotrya* and used seven species of *Eriobotrya* and two- related species to conduct 91 cross combinations between *Eriobotrya* plants and 21 cross combinations between *Eriobotrya* plants and related plants from 2004 to 2014 (Lin, 2017). Previous studies found that the fruit of the interspecific hybrid JT8 obviously had a granular taste similar to that of pear fruits (unpublished). In this study, we found that some stone cell traits were significantly different among *Eriobotrya* plants. The key finding of this study is that stone cell traits could be transmitted from parents to progenies in interspecific crosses, and the transgressive inheritance of stone cell traits might occur. Therefore, it is feasible to transfer stone cell traits of wild loquat into common loquat cultivars by cross-breeding. The content of pear stone cells exhibited quantitative trait inheritance, and the content of pear stone cells in hybrid offspring showed a trend of significant increase compared with their parents (Cui et al., 2011; Zhang et al., 2018). It has also been reported that the content of stone cells in hybrid offspring tended to be distributed around the mid-parent value, and the contents of stone cells were greatly affected by the male parent (Bai et al., 2018). The size and density of pear SCCs also showed quantitative trait inheritance, and the size and density of the hybrid offspring tended to be larger and smaller than those of the parent, respectively. The degree of variation was different from those of the parental varieties (Cui et al., 2011; Zhang et al., 2018). The propensity for the inheritance of pear stone cells could be used to partly explain the stone cell traits of the interspecific hybrid JT8 in this study, but determining whether the inheritance of stone cell traits in the loquat interspecific hybrid population is similar to that of pear requires further study.

Compared with apple, pear, and many other *Eriobotrya* fruits, loquat fruits have poor storage and transport tolerance, which is one of the problems that the industry urgently needs to solve (Lin et al., 2018). A few reports have revealed a certain correlation between stone cell traits and storage and transport tolerance in loquat cultivars (Lin et al., 2009; Zhu et al., 2018). We further verified the relationship between stone cell traits and storage and transport tolerance in loquat fruits and found that there were few stone cells during the whole fruit development process in a storage- and transport-susceptible cultivar. This suggested that stone cell traits have potential application value to improve the storage and transport tolerance of loquat cultivars. However, all of the main loquat cultivars were domesticated from a species of *Eriobotrya* named common loquat, whose genetic basis was very narrow (Wang and Lin, 2012). In contrast, the genetic basis of pear cultivars is much broader, and at least five species of pear plants are ancestors of domesticated cultivars, including *Pyrus ussuriensis*, *P. sinkiangensis*, *P. pyrifolia*, *P. communis*, and *P.* x *bretschneideri*. The stone cell traits of these cultivars and their interspecific hybrids showed abundant diversity (Cao et al., 2010; Tian et al., 2011). Stone cell traits, such as content, size, and density, can affect the quality of pear fruits, so one of the main goals of pear breeding is to breed excellent varieties with fewer stone cells (Choi and Lee, 2013; Zhang et al., 2017, 2018). Nevertheless, the results of this study showed that the content and density of stone cells and the diameter of SCCs in loquat cultivars were relatively small. Therefore, it was necessary to use interspecific hybridization to transfer appropriate stone cell traits from wild loquat into loquat cultivars, thus improving the storage and transport tolerance of loquat cultivars.

JFZ was tolerant to storage and transport, while BL was susceptible to these factors. However, the pulp texture was coarse in the JFZ and very fine in the BL (Lin et al., 2008). Both JFZ and BL are regarded as “standard” varieties of “fruit hardness” and other fruit traits in the Chinese DUS test standard of loquat (Lin et al., 2019). In China, breeding research attention has traditionally been placed on maintaining the original taste of loquat by ensuring a high TSS content, with storage and transport tolerance only being considered in recent years. Our results suggested that higher stone cell content and density might be beneficial to enhance storage and transport tolerance while worsening the taste of loquat fruits. Therefore, it will be necessary to investigate the correlations between storage and transport tolerance and fruit texture and taste in loquat to ensure a good balance between these characteristics in future breeding programs.

Different pear varieties exhibit diverse stone cell traits. A large diameter and density of SCCs lead to a more crude and granular taste. The effect of SCCs with diameter greater than 250 μm on pulp texture is significant (Cao et al., 2010; Tian et al., 2011). In this study, although the size of TL stone cells was small, the stronger aggregation of TL stone cells led to a larger size of SCCs in this species than in JFZ. However, JT8 SCCs combined the aggregation traits from the male parent TL and the size traits from the female parent JFZ, thereby exhibiting transgressive inheritance for the SCC size-related traits. The length and width of most JFZ SCCs were less than 250 μm. In contrast, the length of most SCCs and the width of more than half of the SCCs were greater than 250 μm in JT8, and certain SCCs could exceed 1,000 μm in length and width. This may be the reason why JT8 fruits had an obviously granular taste, while JFZ fruits did not. Therefore, efforts to improve the storage and transport tolerance of loquat cultivars by using stone cell traits from wild loquat should fully consider the effects of stone cell traits on the taste of interspecific hybrid fruits. At the same time, a common disadvantage of interspecific hybrids is that their fruits are usually smaller than those of loquat cultivars (e.g., JFZ); therefore, backcrossing is essential (Lin, 2017). In addition, it is worth noting that there was high stone cell content in the peels of pear fruits, and the distribution density of stone cells was as follows: within the peel > in the pulp near the core > in the pulp near the peel (Zhang et al., 2017). Stone cells have high mechanical strength and could fulfill a supporting function and even a role in physical defense against insect herbivory (Whitehill et al., 2016). In this study, no obvious stone cells could be found in the peel tissues of three *Eriobotrya* plants, which we considered to be one of the important reasons why loquat fruits were susceptible to storage and transport tolerance. It is not clear whether there are wild loquats with stone cells distributed in the peels. Therefore, the fruit stone cell traits in wild loquat need to be further studied. Wild loquat with desirable stone cell traits (e.g., content and size or distribution in fruit peels) will be beneficial for improving the storage and transport tolerance of loquat cultivars by using these traits.

## Conclusion

Overall, the shape, size, development, and distribution dynamics of stone cells of *Eriobotrya* plants were thoroughly studied. It was found that the stone cell traits of *Eriobotrya* plants were highly similar to those of pear, indicating a relationship between loquat and pear, which may provide a new idea for the study of the relationship and evolution among the fruit trees of *Rosaceae*. Moreover, the results also demonstrated that stone cell traits could be transmitted from parents to progenies in interspecific crosses. Thus, it is feasible to transfer stone cell traits of wild loquat into common loquat cultivars by cross-breeding. Our results provide a new approach to improving the storage and transport tolerance of loquat cultivars through the use of stone cell traits from wild loquat plants.

## Data Availability Statement

The raw data supporting the conclusions of this article will be made available by the authors, without undue reservation.

## Author Contributions

JW designed and supervised the experiment. SL and DL mainly performed the research and drafted the manuscript. BW and SM carried out the statistical data analysis. SS, TZ, WZ, YB, and QW finished specific parts of the experiments. JW and SL revised the manuscript. All authors contributed to the article and approved the submitted version.

## Funding

This research was funded by the Natural Science Foundation of Fujian Province (2019J01809 and 2021J05240), the Fujian Provincial Science and Technology Project (2021N5014), the Education and Research Project of Young and Middle-Aged Teachers of Fujian Province (JAT170501 and JAT200524), the Research Project of Putian Science and Technology Bureau (2018ZP08 and 2020NP001), the Research and Innovation Special Foundation of Putian University (2016CX001), and the Scientific Research Project of Putian University (2016069 and 2018064).

## Conflict of Interest

The authors declare that the research was conducted in the absence of any commercial or financial relationships that could be construed as a potential conflict of interest.

## Publisher’s Note

All claims expressed in this article are solely those of the authors and do not necessarily represent those of their affiliated organizations, or those of the publisher, the editors and the reviewers. Any product that may be evaluated in this article, or claim that may be made by its manufacturer, is not guaranteed or endorsed by the publisher.
